# Relationship between abnormal pelvic floor electromyography and obstetric factors in postpartum women: a cross-sectional study

**DOI:** 10.1186/s12905-024-03045-8

**Published:** 2024-04-15

**Authors:** Juan Jiang, Chun Li, He-Yu Liu, Zheng-Yan Zhu

**Affiliations:** Department of Obstetrics and Gynecology, Tongren Hospital of WuHan University (Wuhan Third Hospital), No. 216, Guanshan Avenue, Hongshan District, Wuhan, 430014 China

**Keywords:** Cesarean section, Delivery, Glazer protocol, Vaginal birth, Obstetric characteristics, Pelvic floor disorders (PFDs), Surface electromyography (sEMG)

## Abstract

**Objective:**

To evaluate the surface electromyography (sEMG) of pelvic floor muscles (PFMs), compare between vaginal birth and cesarean section and correlate with maternity and obstetrics characteristics in primiparous 6–8 weeks postpartum.

**Methods:**

PFMs surface electromyography screening data of primiparous postpartum women in our hospital at 6–8 weeks postpartum from 2018 to 2021 were selected and analyzed. The study collected data on delivery activities of 543 postpartum women totally.

**Results:**

In general, the abnormal incidence of pelvic floor electromyography in postpartum women mainly occurred in slow muscle (type I fiber) stage and endurance testing stage. Compared to vaginal birth postpartum women, the incidence of abnormal pelvic floor electromyography in cesarean section postpartum women is lower. There were statistical differences in measurement values of pelvic floor electromyography in several different stages between cesarean section and vaginal birth (*P* < 0.005). Regarding the influence on pelvic floor electromyography, there were more influencing factors on vaginal birth postpartum women including age, height, weight, weight gain during pregnancy, gestational week, and first and second stage of labor than on cesarean section postpartum women whose influencing factors included age, weight gain during pregnancy, and newborn weight.

**Conclusion:**

Effects on surface electromyography (sEMG) of pelvic floor muscles (PFMs) at 6–8 weeks postpartum differed based on the different modes of delivery. The high-risk obstetric factors closely related to abnormal surface electromyography (sEMG) of pelvic floor muscles (PFMs) were maternal age, height, weight, and second stage of labor.

## Introduction

As one of the important factors determining the quality of women’s lives, female pelvic floor muscles (PFMs) have been closely linked to physiological activities such as supporting internal organs, controlling urination and defecation, body position, and exercise, as well as play a huge role in sexual intercourse and childbirth. Pelvic floor disorders (PFDs) mainly involve pelvic floor-related muscle dysfunctions or diseases, with the most common cause of disorders being PFMs function degeneration or damage. At present, the commonly used holistic assessment methods of PFMs function include manipulation evaluation, vaginal pressure measurement, POP-Q scale assessment, transperineal PFMs ultrasonography, pelvic floor magnetic resonance imaging (MRI), pelvic floor surface electromyography (sEMG), etc [1]. Typically, sEMG is characterized as a real-time, non-invasive, reliable test that has a good correlation with resting tension measurement using manometry, maximum voluntary contraction (MVC) and PFMs endurance, reflecting muscle activation and fatigue, etc [2]. Currently, the commonly used evaluation protocol worldwide is the Glazer pelvic floor electromyography evaluation protocol proposed by Glazer and Marinsff. This provides a fixed model for measuring PFMs activity, and establishes a database of pelvic floor electromyography descriptions for normal people and those with PFDs [3].

PFDs are often associated with childbirth in women, with a higher incidence among those who have undergone vaginal delivery than cesarean Sects. [4–6]. According to Kuhlmann et al., the absolute risks of stress urinary incontinence (SUI) and pelvic organ prolapse (POP) after cesarean section are estimated to be 7% and 5%, respectively, and 13% and 14% after vaginal delivery, respectively [7]. However, we still don’t know much about the relationship between exposure of various obstetric delivery activities as well as the course and progress of pelvic floor diseases in women’s lives. Early identification of PFDs after delivery-related trauma enables us to effectively strategize and focus on pelvic floor rehabilitation to clinically interfere with and prevent the subsequent aggravation of PFDs requiring surgery. Moreover, the identification and prevention of long-term morbidity caused by pelvic floor injuries will also reduce medical costs [6].

This study aims to describe abnormal postpartum pelvic floor electromyography and determine the characteristics of maternity and obstetrics related to abnormal pelvic floor electromyography in the first 6–8 weeks postpartum.

## Materials and methods

### Study Design

**Fig. 1:**
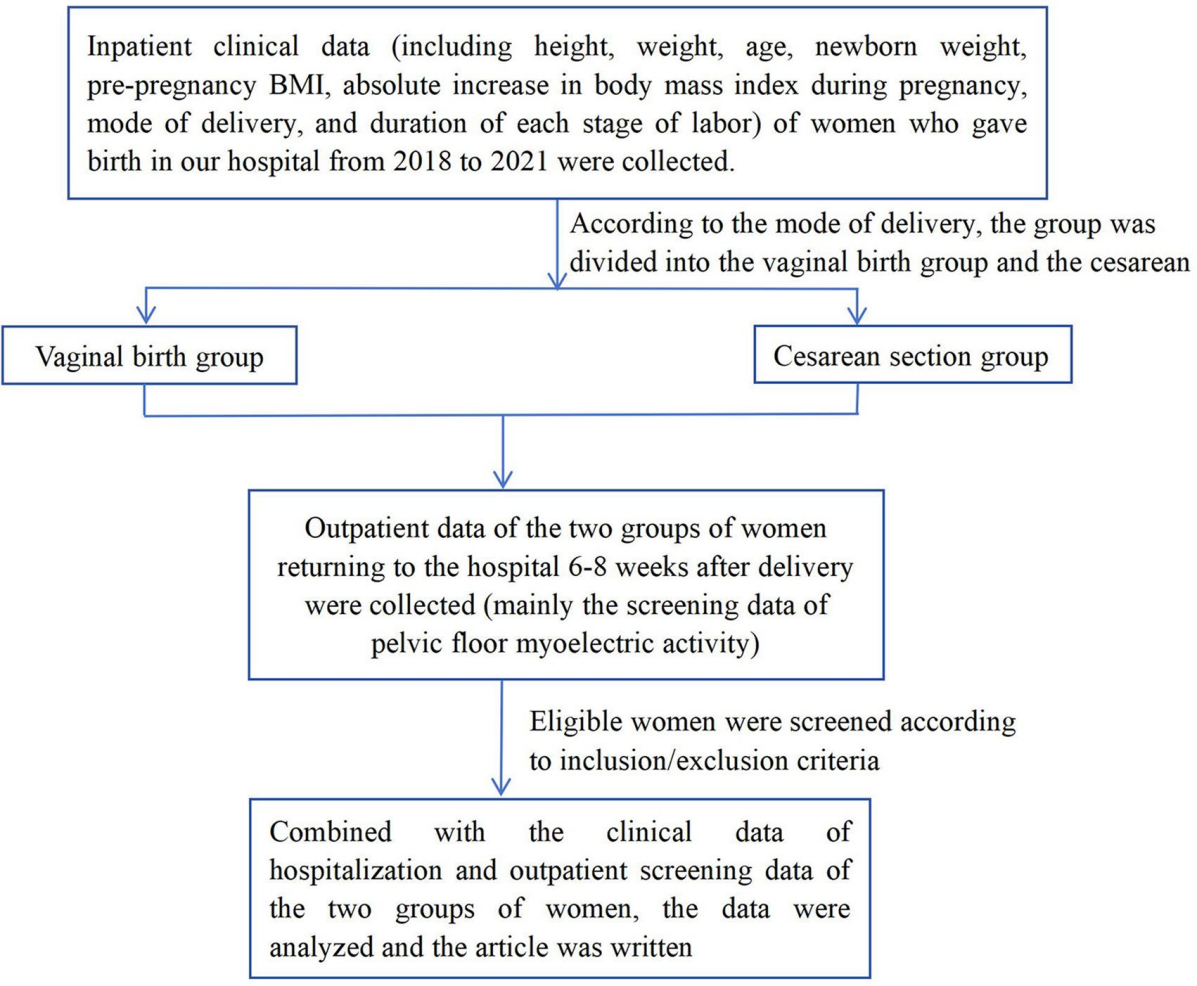
Flow chart of research experiment

This hospital-based, observational, cross-sectional study was conducted among postpartum women who had delivered in Tongren Hospital of Wuhan University in China from 2018 to 2021. Data related to delivery activities from a total of 543 postpartum women were collected. In the actual work, a total of 756 cases of relevant data were collected. According to the inclusion and exclusion criteria, 543 cases of data were adopted, and 213 cases of data were excluded. All the pregnant women who met the inclusion criteria had completed the follow-up. The flowchart can be found in Fig. 1. All participants gave oral and written consent to participate in the study, which was approved by the Ethics Committee of Tongren Hospital of Wuhan University. This study was approved by our hospital’s Medical Ethics Committee.

### Participants

Inclusion criteria were as follows: primipara aged 20–40 years, alive singleton, non-instrumental delivery, suffering from no vaginal bleeding during the evaluation. Relevant sociological and obstetric factors including newborn birth weight, antenatal BMI of the postpartum women, absolute value of BMI rise during pregnancy, delivery modes, duration of labor, were collected at 6–8 weeks postpartum. Exclusion criteria were as follows: severe vaginal or rectal infection, positive pregnancy test, menstrual period, stress/urge urinary incontinence, and pelvic floor organ prolapse ≥ 2 grade (uterus, cystocele, and rectocele during valsalva movement). Based on delivery modes, postpartum women were divided into cesarean section group and vaginal birth group.

### Procedures

The pelvic floor electromyography analysis system (Medlander, MLD A2, Nanjing) consisting of a host computer, a liquid crystal display, electrode wires, disposable vaginal electrodes, body surface electrodes for physiotherapy, etc., was used for detection. To achieve standardization, an experienced therapist instructed on the correct activation of PFMs before the test, and observed whether patients used abdominal, perineal, hip, and gluteal muscles to cause stress at the same time. This ensured that the participants could accurately contract and individually activate PFMs [8]. After they received the instructions and were prepared, the pelvic floor surface electromyographic activity was evaluated using the Glazer protocol, which has five stages: pre-baseline, fast muscle contraction, slow muscle contraction, endurance contraction, and post-baseline.

(1) Pre-baseline stage: Measurement indexes include average mean amplitude (RMS, µV) and variability. This stage helps in the evaluation of resting state of PFMs in the initial stage.

(2) Fast muscle contraction stage: Measurement indexes include maximal peak amplitude (RMS, µV), rising time (s) and falling time (s) of fast muscle. This stage is used to evaluate the contractility of class II muscle fibers of PFMs and the reaction speed of muscle fiber recruitment.

(3) Slow muscle contraction stage: Measurement indexes include average mean amplitude (RMS, µV), variability, rising time (s) and falling time (s) of slow muscle contraction. This stage is used to evaluate the contractility of class I muscle fibers of PFMs and the reaction speed of muscle fiber recruitment.

(4) Endurance contraction stage: Measurement indexes include average muscle strength (RMS, µV) and variability of endurance contraction. This stage involves evaluation of endurance muscle fiber testing, which helps in determining the types of muscle fibers involved in sustained endurance contraction. This stage also helps us understand the effects of those contractions on resting potential and muscle fatigue during endurance contraction.

(5) Post-baseline stage: Measurement indexes include average mean amplitude (RMS, µV) and variability. This stage is used to evaluate the ability of PFMs to return to resting state after undergoing a series of exercises.

After the above activities were completed, the total score was calculated by weighted average of the segmented scores received in each stage, where a total score ≥ 80 was defined as normal, 70–80 as mild; 60–70 as moderate, and < 60 as severe.

### Statistical analysis

We executed SPSS 25. 0 to perform statistical analysis. Here, we compared the means using an unpaired two-tailed Student t-test. Abnormal distribution or variance of quantitative data was described by median ± standard deviation, and compared using Kruskal-Wallis rank sum test. The linear relationship between quantitative data and their correlation was evaluated using Pearson’s correlation analysis. The assumed values of correlation coefficient range from negative correlation (-1) to uncorrelated (0) to positive correlation (+ 1). The symbol of correlation coefficient (i.e., positive or negative) determined the direction of the relationship. If *P* < 0.05, it was considered to be statistically significant.

## Results

A total of 543 patients, including 223 cesarean section and 320 vaginal birth primiparous postpartum women were enrolled in this study. Table 1 shows the demographic characteristics such as age, height, weight, of postpartum women at 6–8 weeks postpartum in this study. Table 2 showed the statistical characteristics of the obstetric factors such as newborn birth weight, antenatal BMI of the postpartum women, absolute value of BMI rise during pregnancy, delivery modes, duration of labor, of postpartum women at 6–8 weeks postpartum in this study. The demographic characteristics and obstetric factors of patients were matched by t-test; there was no significant difference in mean age, height, weight, newborn birth weight, antenatal BMI of the postpartum women, absolute value of BMI rise during pregnancy between cesarean section and vaginal delivery.

**Table 1:** **Demographic characteristics of postpartum women at 6–8 weeks postpartum (mean ± standard deviation, [95% confidence interval])**

Group	Age (years)	Height (cm)	Weight (kg)
Overall (*n* = 543)	27.56 ± 2.76 [27.328–27.792]	160.38 ± 5.09 [159.952-160.808]	66.68 ± 8.50 [65.965–67.395]
Cesarean section (*n* = 223)	28.37 ± 2.81 [28.001–28.739]	159.94 ± 5.18 [159.260-160.620]	66.97 ± 9.34 [65.744–68.196]
Vaginal birth (*n* = 320)	27.74 ± 2.55 [27.461–28.019]	160.33 ± 10.02 [159.232-161.428]	66.70 ± 7.68 [65.859–67.541]

**Table 2:** **Statistical characteristics of the obstetric factors of postpartum women at 6–8 weeks postpartum(mean ± standard deviation, [95% confidence interval])**

Group	Newborn birth weight(g)	Antenatal BMI of the postpartum women	Absolute value of BMI rise during pregnancy	First stage of labor(min)	Second stage of labor(min)	Total stage of labor(min)
Overall (*n* = 543)	3250 ± 416.70 [3208.907-3279.093]	26.265 ± 3.001 [26.012–26.518]	5.69 ± 1.700 [5.547–5.833]	N/A	N/A	N/A
Cesarean section (*n* = 223)	3304 ± 474.25 [3241.414-3366.586]	26.950 ± 3.289 [26.516–27.384]	5.66 ± 1.898 [5.41–5.910]	N/A	N/A	N/A
Vaginal birth (*n* = 320)	3203 ± 365.73 [3162.776-3243.224]	25.784 ± 2.691 [25.488–26.080]	5.72 ± 1.560 [5.548–5.892]	430 ± 209.287 [406.982-453.018]	37 ± 31.398 [33.547–40.453]	477.50 ± 220.181 [453.284-501.716]

**Fig. 2:**
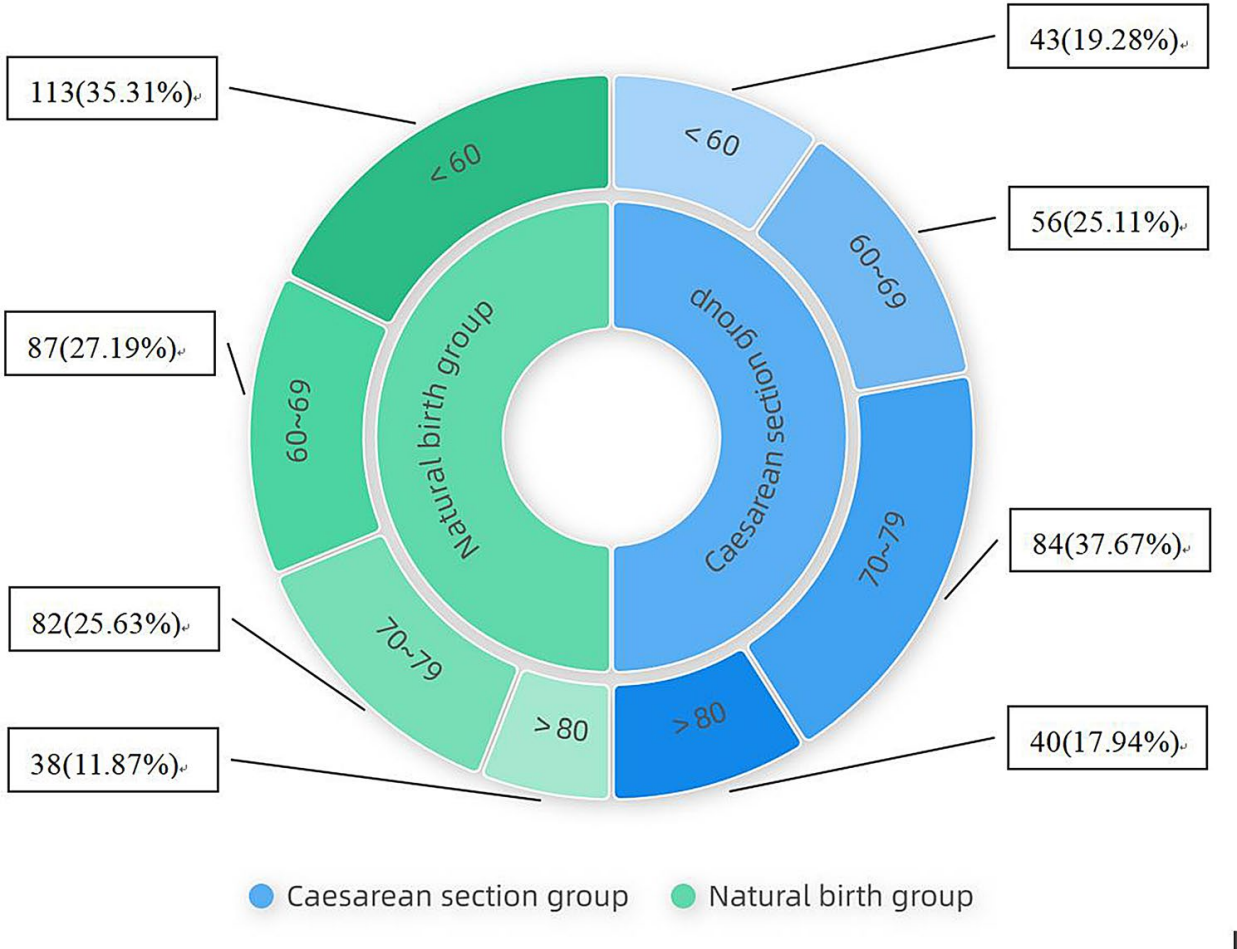
sEMG total score distribution of postpartum women with different delivery modes (n%)

The sEMG measurement values of all 543 patients at 6–8 weeks postpartum were recorded. As shown in Fig. 2, the population proportion of total sEMG scores of cesarean section postpartum women were as follows in descending order: 70–79 (37.67%), 60–69 (25.11%), < 60 (19.28%), > 80 (17.94%), while that of vaginal birth postpartum women were as follows in descending order: < 60 (35.31%), 60–69 (27.19%), 70–79 (25.63%), > 80 (11.87%). It is evident that there are significant differences in pelvic floor electromyography activity of postpartum women for different delivery modes at 6–8 weeks postpartum. As shown in Table 3, the population distribution characteristics of sEMG measurement of postpartum women with different delivery modes at 6–8 weeks postpartum were as follows: Significant proportion of abnormal pelvic floor electromyography (n% ≥ 70%) of postpartum women in general mainly occurred during the slow muscle (type I fiber) stage (*n* = 419, n% = 77.16%) and endurance stage (*n* = 402, n% = 74.03%). The abnormal pelvic floor electromyography of cesarean section patients mainly occurred during the pre-baseline stage (*n* = 160, n% = 71.75%), while that of vaginal delivery patients was during the slow muscle (type I fiber) stage (*n* = 270, n% = 84.38%) and endurance stage (*n* = 255, n% = 79.69%).

**Table 3:** Population distribution characteristics of sEMG measurement of postpartum women at 6–8 weeks postpartum

Stage		Standard value	Total postpartum women (n%)	Cesarean section postpartum women (n%)	Vaginal delivery postpartum women (n%)
Pre-baseline stage	Average	(< 4uv)	319(58.75%)	160(71.75%)	159(49.69%)
	Variability	(< 0.2)	160(29.47%)	65(29.15%)	95(29.69%)
Fast muscle (type II fiber) stage	Maximum	(> 40uv)	330(60.77%)	117(52.47%)	213(66.56%)
	Rising time	(< 0.5s)	253(46.59%)	96(43.05%)	157(49.06%)
	Recovery time	(< 0.5s)	326(60.04%)	127(56.95%)	199(62.19%)
Slow muscle (type I fiber) stage	Average	(> 35uv)	419(77.16%)	149(66.82%)	270(84.38%)
	Variability	(< 0.2)	318(58.56%)	112(50.22%)	206(64.38%)
Endurance stage	Average	(> 30uv)	402(74.03%)	147(65.92%)	255(79.69%)
	Variability	(< 0.2)	296(54.51%)	104(46.64%)	192(60.00%)
	Last/first 10 s ratio	(0.8–1.2)	124(22.84%)	42(18.83%)	106(33.13%)
Post-baseline stage	Average	(< 4uv)	237(43.65%)	118(52.915)	117(36.56%)
	Variability	(< 0.2)	167(30.76%)	70(31.39%)	98(30.62%)

PS: Definition of abnormal pelvic floor sEMG data: lower or higher than the corresponding standard value

Median ± standard deviation of sEMG measurement in cesarean section and vaginal birth patients are displayed in Table 4; they were compared using unpaired t-test in each stage. Statistically significant differences were noted in the measurement values of pelvic floor electromyography between cesarean section and vaginal delivery (*P* < 0.005), except for the rising time (*P* < 0.171) and recovery time (*P* < 0.175) of fast muscle (type II fiber) stage as well as ratio of first 10 s and last 10 s of endurance stage (*P* < 0.979). Compared to vaginal delivery postpartum women, the average values during the pre-baseline and post-baseline stages were higher than that of cesarean section postpartum women, resulting in lower total scores in these two stages. However, for vaginal delivery postpartum women, the scores of pre-baseline (3.6 ± 3.267) and post-baseline (2.3 ± 5.592) stages were higher, indicating the relative advantages in static PFMs tension and muscle recovery ability. The maximum value of fast muscle (type II fiber) stage, average value of slow muscle (type I fiber) stage, and average value of endurance stage in both groups were lower than normal, reflecting the downward trend of muscle strength and endurance of fast muscle (type II fiber) and slow muscle (type I fiber) at 6–8 weeks postpartum. Among these, the weakness of vaginal delivery postpartum women was more obvious, showing statistically significant differences from the cesarean section postpartum women (*P* = 0.000).

**Table 4:** Comparison of pelvic floor electromyography data (median ± standard deviation, [95% confidence interval]) between cesarean section postpartum women and vaginal birth postpartum women at 6–8 weeks postpartum

Stage	Group	Standard value	Cesarean section group (*n* =223)	Vaginal birth group (*n* =320)	P
Pre-baseline stage	Average	(< 4uv)	6.5 ± 4.077 [5.965–7.035]	3.6 ± 3.267 [3.242–3.958]	***
	Variability	(< 0.2)	0.16 ± 0.164 [0.138–0.182]	0.17 ± 0.371 [0.129–0.211]	***
	Partial score	-	45 ± 26.843 [41.477–48.523]	75 ± 24.216 [72.347–77.653]	***
Fast muscle (type II fiber) stage	Maximum	(> 40uv)	39.2 ± 18.087 [36.826–41.574]	31.4 ± 16.966 [29.541–33.259]	***
	Rising time	(< 0.5s)	0.47 ± 0.218 [0.441–0.499]	0.5 ± 0.251 [0.472–0.528]	*P* < 0.171
	Recovery time	(< 0.5s)	0.55 ± 0.313 [0.509–0.591]	0.56 ± 0.441 [0.512–0.608]	*P* < 0.175
	Partial score	-	81 ± 15.996 [78.901–83.099]	75 ± 22.981 [72.482–77.518]	***
Slow muscle (type I fiber) stage	Average	(> 35uv)	30.2 ± 13.14 [28.475–31.925]	22.5 ± 12.68 [21.111–23.889]	***
	Variability	(< 0.2)	0.21 ± 0.108 [0.196–0.224]	0.24 ± 0.142 [0.224–0.256]	***
	Partial score	-	72 ± 17.338 [69.724–74.276]	58 ± 21.288 [55.668–60.332]	***
Endurance stage	Average	(> 30uv)	26.3 ± 11.832 [24.747–27.853]	18.4 ± 11.32 [17.160–19.640]	***
	Variability	(< 0.2)	0.2 ± 1.245 [0.037–0.363]	0.23 ± 0.13 [0.216–0.244]	**
	Last/First 10 s ratio	(0.8–1.2)	0.97 ± 0.194 [0.945–0.995]	0.96 ± 0.554 [0.899–1.021]	*P* < 0.979
	Partial score	-	80 ± 14.368 [78.114–81.886]	69 ± 17.487 [67.084–70.916]	***
Post-baseline stage	Average	(< 4uv)	4.4 ± 3.579 [3.930–4.870]	2.3 ± 5.592 [1.687–2.913]	***
	Variability	(< 0.2)	0.16 ± 0.167 [0.138–0.182]	0.2 ± 0.382 [0.158–0.242]	***
	Partial score	-	70 ± 25.892 [66.602–73.398]	83 ± 24.187 [80.350–85.650]	***
	Total score	-	71.5 ± 12.236 [69.894–73.106]	66.5 ± 14.256 [64.938–68.062]	***

*Note* (1) ***, **, and * represent the significance of 1%, 5%, and 10% respectively;

To explore the abnormal pelvic floor electromyography at 6–8 weeks postpartum, the correlation between the following variables and sEMG scores in five stages was analyzed using Pearson’s correlation analysis. We assumed that abnormal behaviors were related to several factors in childbirth, and collected related data of 223 cesarean section postpartum women and 320 vaginal birth postpartum women including age (28 ± 2.80 years), height (160 ± 7.50 cm), weight (67 ± 8.5 kg), weight gain during pregnancy (15 ± 4.50 kg), gestational weeks at delivery (39 ± 2.50 weeks), newborn birth weight (3250 ± 416.70 g), first stage of labor (430 ± 209.287 min), second stage of labor (37 ± 31.398 min) and total stage of labor (477.50 ± 220.181 min). Missing data were rejected. Detailed data are described in S1-2 text.

**Fig. 3:**
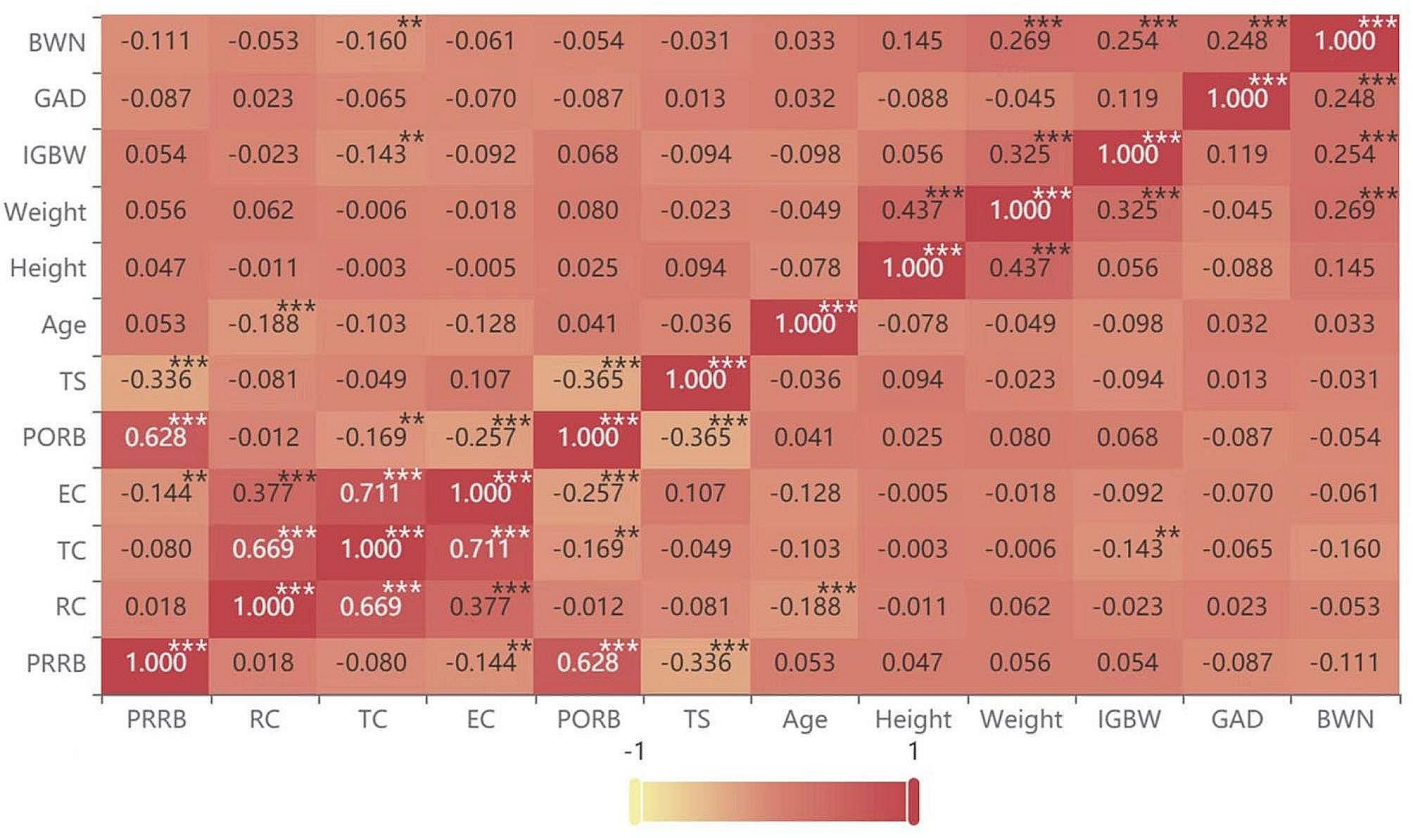
Pearson’s partial correlation analysis results of related variables of cesarean section postpartum women at 6–8 weeks postpartum.*** and ** represent the significance of 1% and 5%, respectively

**Fig. 4:**
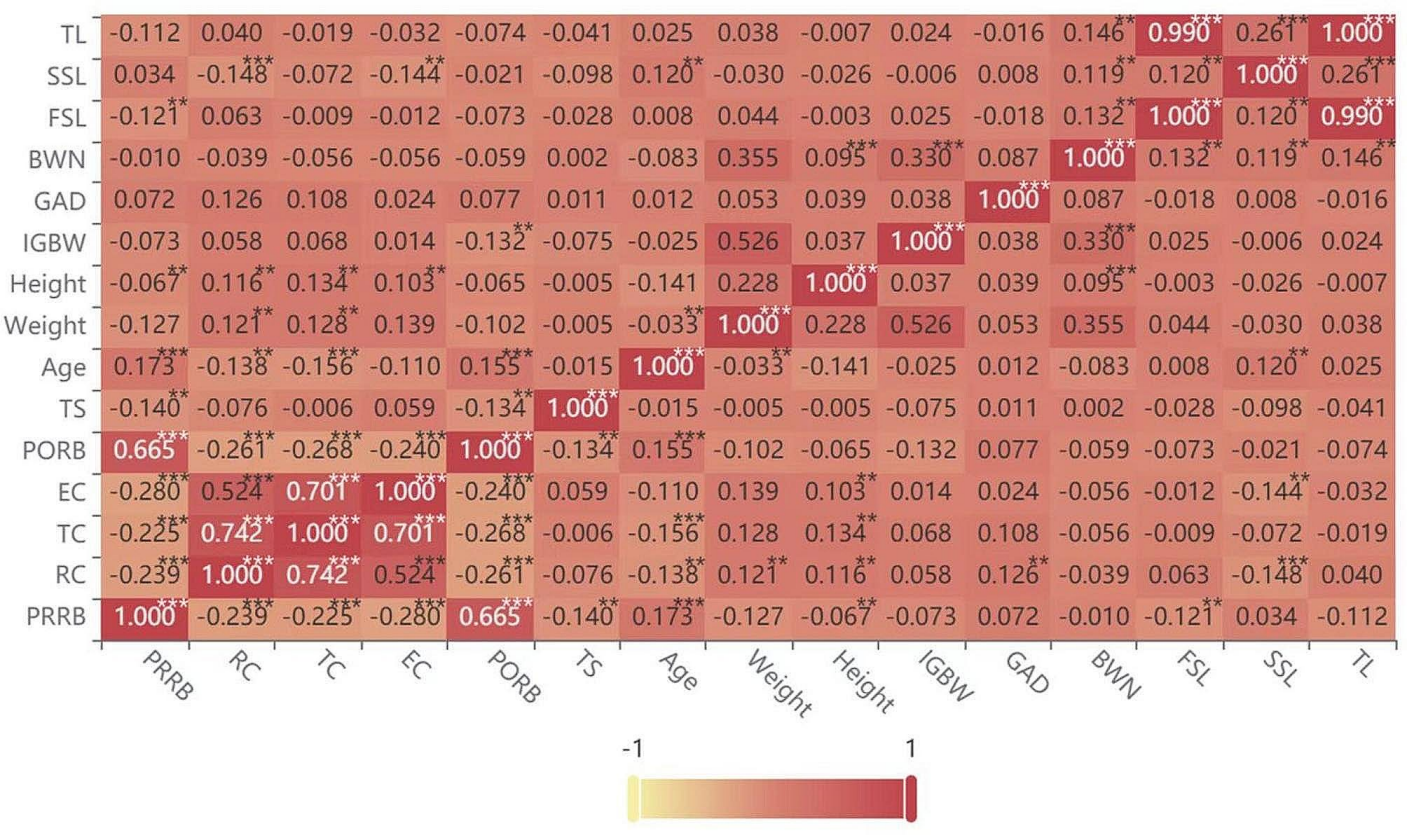
Pearson’s partial correlation analysis results of related variables of vaginal birth postpartum women at 6–8 weeks postpartum

Pearson’s correlation coefficients among various variables related to delivery activities of cesarean section or vaginal birth postpartum women at 6–8 weeks postpartum are shown in Figs. 3 and 4, respectively. If the correlation coefficient is closer to 1, the correlation between the two variables is higher. Referring to the influence on pelvic floor electromyography, there were more influencing factors for vaginal birth postpartum women including age, height, weight, weight gain during pregnancy, gestational week, and first and second stage of labor than in cesarean section postpartum women, which included age, weight gain during pregnancy, and newborn weight. The results showed that the slow muscle (type I fiber) stage acquired the highest positive correlation with the endurance stage (r_cesarean section_=0.711, r_vaginal birth_=0.701, *P* = 0.000), followed with high positive correlation between fast muscle (type II fiber) stage and slow muscle (type I fiber) stage (r_cesarean section_=0.669, r_vaginal birth_=0.742, *P* = 0.000). There was also a certain correlation between fast muscle (type II fiber) stage and endurance stage (r_cesarean section_=0.377, r_vaginal birth_=0.524, *P* = 0.000). High positive correlation also occurred between pre-baseline stage and post-baseline stage (r_cesarean section_=0.628, r_vaginal birth_=0.665, *P* = 0.000). In addition, maternal age was negatively correlated to fast muscle (type II fiber) stage (*r*_*cesarean section*_=-0.188, r_vaginal birth_=-0.138, *P* < 0.05) and slow muscle (type I fiber) stage of vaginal birth postpartum women (r_vaginal birth_ =-0.156, *P* < 0.05), and was positively correlated to pre-baseline stage (r_vaginal birth_ =-0.173, P *<* 0.05) and post-baseline stage (r_vaginal birth_ =-0.155, *P* < 0.05) of vaginal birth postpartum women. Height was positively correlated with fast muscle (type II fiber) stage (r_vaginal birth_ =0.116, *P* < 0.05) and slow muscle (type I fiber) stage (r_vaginal birth_=0.134, *P* < 0.05) of vaginal birth postpartum women. Weight was positively correlated with fast muscle (type II fiber) stage (r_vaginal birth_ =0.121, P *<* 0.05), slow muscle (type I fiber) stage (r_vaginal birth_ =0.128, *P* < 0.05) and endurance stage (r_vaginal birth_ =0.139, *P* < 0.05) of vaginal birth postpartum women and was negatively correlated to pre-baseline stage (r_vaginal birth_=-0.127, P *<* 0.05). The second stage of labor was negatively correlated to fast muscle (type II fiber) stage (r_vaginal birth_ =-0.148, *P* = 0.01) and endurance stage (r_vaginal birth_ =-0.144, *P* < 0.05) of vaginal birth postpartum women.

## Discussion

The incidence of PFD is complex and closely related to the effects of delivery mode, obesity during pregnancy, chronic injury, and uneven distribution of hormones on the nutritional supply of pelvic floor tissues [9]. So far, delivery and pregnancy are considered to be the main reasons. Excessive stretching during delivery and pregnancy weakens the elasticity of fascia and muscle fibers and induces muscle fiber tearing, leading to prolapse of anterior and posterior vaginal walls and destruction of pelvic floor support structures. For women to recover to normalcy in the early postpartum period, the active healing of muscles, ligaments, and nerves and their regeneration period naturally take time. Therefore, it is of immense clinical significance to identify the risk factors of pelvic floor dysfunction in a timely manner for early prevention and treatment of diseases and encourage maternal recovery.

In this study, we found the potential relationship between the five stages of sEMG. Interestingly, among these five stages of vaginal birth postpartum women, negative linear correlations occurred in pre-baseline and post-baseline stage in three stages [fast muscle (type II fiber) stage, slow muscle (type I fiber) stage and endurance stage]. This suggests that the greater the resting tension of PFMs, the worse the muscle strength and endurance of fast and slow muscle fibers. Potential causes are yet to be further studied. In addition, results showed significant differences in the effects of different delivery modes on pelvic floor electromyographic activities at 6–8 weeks postpartum based on female obstetric characteristics. As confirmed using sEMG, the incidence of abnormal pelvic floor electromyography in cesarean section postpartum women was lower than in vaginal birth postpartum women. Compared to vaginal delivery postpartum women, the average values in pre-baseline and post-baseline stages were higher in cesarean section postpartum women, resulting in lower total scores for these two stages. This indicates higher static PFMs tension at 6–8 weeks postpartum and weak muscle activity stability and recovery ability. In previous studies, this was demonstrated to easily cause PFMs spasms, wherein muscles cannot fully relax or contract effectively, potentially leading to chronic pelvic pain and constipation [10]. This phenomenon was confirmed by Baud [4], with the potential causes related to the specific position of the cesarean section incision. Common suprapubic scars can entangle the ilioinguinal or iliohypogastric nerves, leading to diffuse persistent neuropathic pain [11], and more samples would be required to confirm using experiments and studies. In addition, PFMs are composed of 70% slow muscle fibers, which provide long-term basic tension and support pelvic organs to stay upright against gravity. In this study, the sEMG of vaginal birth postpartum women revealed that the scores of fast muscle (type II fiber), slow muscle (type I fiber), and endurance stage were all low, suggesting that postpartum PFMs strength and endurance decreased, especially for slow muscle fibers, which led to weakened pelvic floor support and easy inducement of PFDs [10]. From these results, we deduced that compared to vaginal delivery, the ratio of cesarean section to abnormal pelvic floor electromyography is lower. In addition, there are various studies that show the relationship between cesarean section and the decreased incidence of urinary incontinence and pelvic organ prolapse. However, the indications of cesarean section should still be carefully considered due to its correlation to increased probability of asthma and obesity in children, low fertility in future, and several subsequent pregnancy risks such as placenta previa, uterine rupture, and stillbirth [12].

Furthermore, we also considered a variety of important pregnancy and obstetric risk factors to identify women who were more prone to PFDs. Results showed that maternal age, height, weight, and the second stage of labor, (the definition of which is based on the standard of Chinese primipara) are the high-risk obstetrics factors closely related to abnormal pelvic floor electromyography, while long-term follow-up is needed to confirm the incidence of PFDs in such people.

Future research should focus on whether pelvic floor electromyography measured using sEMG in the early postpartum period can predict the direction of pelvic floor support and symptom load 1 to 3 years after the first delivery, and should also consider whether high-risk factors such as maternal age, height, weight, and the second stage of labor can be potentially used to formulate individualized prevention and treatment programs for PFDs in the early postpartum period.

## Data Availability

The data that support the findings of this study are available from the corresponding author upon reasonable request.
